# Intelligent model for the detection and classification of encrypted network traffic in cloud infrastructure

**DOI:** 10.7717/peerj-cs.2027

**Published:** 2024-05-27

**Authors:** Muhammad Dawood, Chunagbai Xiao, Shanshan Tu, Faiz Abdullah Alotaibi, Mrim M. Alnfiai, Muhammad Farhan

**Affiliations:** 1 Faculty of Information Technology, Beijing University of Technology, Beijing, China; 2 Department of Information Science, College of Humanities and Social Sciences, King Saud University, Riyadh, Saudi Arabia; 3 Department of Information Technology, College of Computers and Information Technology, Taif University, Taif, Saudi Arabia; 4 School of Science and Engineering, Al Akhawayn University in Ifrane, Ifrane, Morocco

**Keywords:** Cloud security, Traffic classification, Intelligent model, Machine learning, SDN

## Abstract

This article explores detecting and categorizing network traffic data using machine-learning (ML) methods, specifically focusing on the Domain Name Server (DNS) protocol. DNS has long been susceptible to various security flaws, frequently exploited over time, making DNS abuse a major concern in cybersecurity. Despite advanced attack, tactics employed by attackers to steal data in real-time, ensuring security and privacy for DNS queries and answers remains challenging. The evolving landscape of internet services has allowed attackers to launch cyber-attacks on computer networks. However, implementing Secure Socket Layer (SSL)-encrypted Hyper Text Transfer Protocol (HTTP) transmission, known as HTTPS, has significantly reduced DNS-based assaults. To further enhance security and mitigate threats like man-in-the-middle attacks, the security community has developed the concept of DNS over HTTPS (DoH). DoH aims to combat the eavesdropping and tampering of DNS data during communication. This study employs a ML-based classification approach on a dataset for traffic analysis. The AdaBoost model effectively classified Malicious and Non-DoH traffic, with accuracies of 75% and 73% for DoH traffic. The support vector classification model with a Radial Basis Function (SVC-RBF) achieved a 76% accuracy in classifying between malicious and non-DoH traffic. The quadratic discriminant analysis (QDA) model achieved 99% accuracy in classifying malicious traffic and 98% in classifying non-DoH traffic.

## Introduction

The unreliable delivery protocol User-Datagram-Protocol (UDP) was used to create the Domain Name System (DNS). The security provided by DNS architecture met all of the Internet’s requirements. This method makes the Internet connection chain susceptible to today’s network protocols since it provides names to address mapping services. New distant weapons, such as cyber strikes, target essential infrastructures, such as presidential campaigns and nuclear programs, government personnel data, and software suppliers are used for cyber-attacks (MontazeriShatoori et al., 2020). When accessing the internet network effectively, it is critical to tell the difference between hazardous and benign data. It is crucial for private networks and the Internet to keep their DNS systems safe from intrusion by unwanted parties. Since hackers exploit advanced strategies to outbreak DNS requests and responses, a covert channel is used to encrypt DNS transfers and queries by establishing a connection with DNS using the HTTPS protocol. Man-in-the-middle attacks are difficult to defend against with this method since they improve privacy and address DNS weaknesses (Banadaki, 2020; Wazan & Cuppens, 2023).

An intrusion detection system (IDS) monitors internet-connected device traffic and detects DoH traffic assaults in network topology by detecting intrusions. Intrusion detection is established by monitoring and analyzing events happening in a computer system or network (Larsen, Pahl & Coatrieux, 2023). The events depend upon the availability or circumvent security safeguards, integrity and efforts to compromise confidentiality. An intrusion detection system (IDS) is your best line of protection against today’s more sophisticated and widespread network assaults. Malicious traffic may be detected and distinguished from legitimate communication using various intrusion detection systems (IDS). Algorithms like naive Bayes, neural network regression, and support vector machines have been used to identify attacks, including principal component analysis, random forest (RF), and support vector machines (Jafar et al., 2021; Vries, 2021).

These methods may test and analyze DoH traffic in covert channels and tunnels. A systematic technique is presented here to evaluate the capabilities of various machine-learning algorithms. This study aims to identify and classify DoH traffic and discriminate between benign and malicious DoH traffic using time-series classifiers in a two-layered ML technique (Raikar et al., 2020). The application of DoH protocol in an application employing four servers and five dissimilar browsers and software applications to record non-DoH, malicious-DoH and benign-DoH traffic is the part of the dataset according to CIC’s current version of their dataset (Banadaki, 2020). Layer one is used to differentiate non-DoH and DoH traffic, while layer two is used to differentiate malicious DoH and benign traffic. Numerous ML methods are being tested to classify between non-DoH and DoH traffic, and in the same way malicious and benign traffic (Khan, Raza & Hwang, 2022; Singh et al., 2022; Singh & Roy, 2020).

In the context of Software Defined Networks (SDN), various approaches exist for detecting DNS tunnels, such as statistical analysis of DNS packets and domain name analysis. These techniques often involve using statistical models to identify anomalous domain names. Indicators of DNS tunnels include DNS resolution frequency, subdomain length, and the presence of TXT records. Strategies like block listing domains, blocking IP addresses, and removing suspicious DNS packets can be employed to mitigate DNS tunneling. SDN, a concept revolutionizing network architecture, plays a crucial role in DNS operations (Jafarian et al., 2021). DNS serves as the backbone of the Internet, translating human-readable hostnames into computer-understandable IP addresses. The development of the DNS protocol followed a decentralized hierarchical approach. When a DNS client initiates a query for an IP address, the local DNS server responds by checking its cache. The query is forwarded to a recursive DNS resolver if the response is not found in the cache. This resolver then iteratively requests information from authoritative name servers, top-level domain (TLD) name servers, and eventually the root name server, until it obtains the authoritative response. DNS tunneling is a technique that leverages the DNS protocol to encapsulate data communication between a client and a server. In this method, data is encoded within the DNS response records of a typical DNS request, and the server may or may not reply with encoded data. By integrating SDN principles into DNS operations, network administrators gain greater control and visibility over DNS traffic. SDN enables centralized management and programmability of network resources, facilitating the implementation of advanced security measures to detect and prevent DNS tunneling attacks.

**Figure 1:**
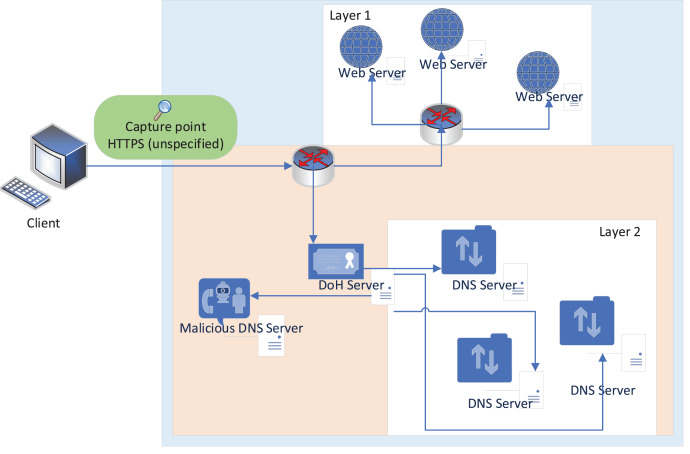
Network topology used to capture the data (MontazeriShatoori et al., 2020).

Capturing DoH and non-DoH traffic is accomplished using a two-layered technique. Browsers that support DoH protocol and DNS tunneling tools are used to visit the top 10,000 Alexa websites and create HTTPS (both benign and malign DoH traffic) and DoH traffic for the representative dataset. A statistical characteristics classifier divides the collected traffic into two categories: DoH and non-DoH. DoH traffic is classified as either benign or malicious at the second layer using a time-series classifier. Accessing a website using the HTTPS protocol generates traffic designated as non-DoH. Many Alexa domain websites are visited to ensure the dataset is well balanced. ‘Benign-DoH’ is non-malicious DoH traffic created using the same method as in ‘non-DoH’ by utilizing the Mozilla Firefox and Google Chrome web browsers’. This is known as malicious DoH traffic and is generated by DNS tunneling software such as dns2tcp, DNSCat2, and Iodine. Using these tools, you may transmit TCP traffic as DNS queries. These programs build encrypted data tunnels, to put it another way. As a result, DNS queries are forwarded to dedicated DoH servers through HTTPS requests encrypting the traffic using TLS (Belel, Dutta & Mukhopadhyay, 2023). Using web browsers, we can simulate normal online behavior, such as utilizing HTTPS and benign DoH. To put it another way, malicious DoH is created using a combination of DoH tunnel-building tools (Khan et al., 2022). This technology’s traffic is logged and used to train the classifiers, as shown in Fig. 1 (MontazeriShatoori et al., 2020).

The researcher explore the application of cloud-based semi-static secure accountable authority identity-based broadcast encryption featuring public traceability without random oracles, in the context of network traffic data detection and categorization using ML methods (Singh, Acharya & Dutta, 2023). The Domain Name Server (DNS), one of the earliest and most vulnerable network protocols, presents numerous security flaws that have been frequently exploited over time, creating significant concern in the realm of cybersecurity. Despite the implementation of sophisticated attack strategies by cyber criminals to pilfer data surreptitiously, ensuring the security and privacy of DNS queries and responses remains a complex task. The ever-evolving landscape of internet services has inadvertently provided a broad playing field for such cyber-attacks on computer networks. They focus on leveraging cloud support to enhance the effectiveness of ML-based classification in network traffic data detection and categorization (Mohamed et al., 2021). The intent is to further fortify the security of DNS communications and mitigate the risk of cyber-attacks, thereby improving the overall security architecture of computer networks.

This article makes three contributions: Firstly, a ML model to differentiate DoH traffic from non-DoH traffic at layer 1. We provide a unique two-layered technique that characterizes DoH traffic at layer 2. Secondly, a labeled dataset may be generated in the network premises by collecting non-DoH encrypted traffic, malicious-DoH and benign-DoH traffic. Thirdly, introducing the notion of packet clumps and illustrating the efficiency of this feature set in encrypted traffic characterization by proposing a new feature set based on time-series representation of traffic flows (Srivastava et al., 2022).

This research makes several unique contributions for detection and classification of DoH network traffic with the application of ML techniques. Firstly, it proposes a novel two-layered classification approach for analyzing DoH communications in depth. At layer one; a statistical characteristic classifier is developed to differentiate DoH traffic from non-DoH traffic. Subsequently, layer two involves classifying the DoH traffic as either benign or malicious using time-series models. Secondly, to facilitate rigorous evaluation of various machine-learning (ML) algorithms, an extensive labeled dataset is carefully generated by collecting samples of benign-DoH, malicious-DoH and non-DoH traffic within a network testbed set-up involving multiple browsers and servers. This provides a robust and representative dataset for comparative assessment. Thirdly, the study introduces the concept of packet clumps as a new feature engineering approach for encrypted traffic analysis. By extracting time-series representations based on packet clump characteristics, this feature set is shown to enhance the effectiveness of ML classifiers for the encryption traffic detection task. Hence, this research advances the state-of-the-art through scientific contributions in multiple dimensions, ranging from a novel classification framework to generation of a benchmark dataset and proposal of improved learning features. The rigorous methodological approach and well-defined contributions allow meaningful evaluation and comparison of ML schemes for DoH network traffic identification and segmentation.

## Literature review

Algorithms LGBM and XGBoost surpass the competition in almost all classification measures, achieving classification task accuracy of 100 percent in layers 1 and 2 (Banadaki, 2020). Source IP was the most important feature for differentiating non-Doh traffic and DoH traffic in layer one, followed by the Destination IP feature, out of 34 characteristics taken from the CIRA-CIC-DoHBrw-2020 dataset. LGBM and gradient boosting techniques use just Destination IP to distinguish benign and malicious data in layer 2 (Alarfaj et al., 2022; Banadaki, 2020). DNS is a critical component of the Internet’s infrastructure. DNS’s main job is to map IP addresses to domain names and send users to the relevant computers, programs, and files (Niakanlahiji et al., 2023; Zang et al., 2023). Because of DNS’s security weaknesses, it is always a prime target for cybercriminals. An attempt to identify fraudulent DNS activity is made using several machine-learning classifiers, including random forest (RF), K-nearest neighbor (KNN), and gradient boosting (GB) (Hadwan et al., 2022; Shiomoto, Otoshi & Murata, 2023; Singh & Roy, 2020; Ullah, Jabbar & Al-Turjman, 2020).

DNS over HTTPS (DoH) improves internet security while enhancing user privacy. DoH, on the other hand, makes it more difficult for network managers to maintain the security of their systems. Because DoH traffic looks like normal HTTPS traffic, it is difficult to tell apart (Khodjaeva & Zincir-Heywood, 2021). DoH network traffic may be distinguished from non-DoH network traffic using many criteria examined in depth in this article (Vries, 2021). DNS is one of the most critical pieces of Internet infrastructure (Mitsuhashi et al., 2021). The proposed scheme’s simulation results suggest that it can distinguish between malicious, benign, and non-DoH classes with a 99 percent accuracy. Many academics have investigated various ML strategies to meet this problem (Waqas et al., 2022). This research presents a systematic technique for recognizing malicious and encrypted DNS requests by monitoring network traffic and determining statistical features (AlQaralleh et al., 2022; Jafar et al., 2021).

The author then adds to these qualities by estimating the flow’s entropy in several methods. Using publicly accessible datasets, the author compares and contrasts five ML classifiers: Decision Tree (DT), RF, Logistic Regression, Support Vector Machine, and Naive Bayes (Khodjaeva & Zincir-Heywood, 2021). Providing improved protection against attacks is becoming more important as the worldwide reach of the Internet of Things (IoT) networks expands annually (Deebak et al., 2022; Tu et al., 2021b; Wang et al., 2021). Cyberattacks may be mitigated most effectively using an IDS (Lehniger, Saad & Langendörfer, 2022). A hybrid lightweight IDS is proposed in this study based on data gathered from IoT networks (Althobaiti et al., 2022; Sarkar et al., 2022; Ullah et al., 2021, 2020). When dealing with a vast dataset, XCNN and RCNN are 1,000 times quicker than KNN. XCNN took 86.18% less time to compute than KNN, but RCNN took 91.74% more. This benefit allows for more latitude in IDS site selection (Tu et al., 2021a). As a result of our IDS’ minimal training requirements, response times to zero-day assaults are cut in half (Alassaf & Sikkandar, 2022; Liu et al., 2021).

### Cloud-based apps security model

Safe instant messaging (IM) protocols should please the broad security areas of confidentiality, reliability, authenticity, and integrity. Few even guarantee cutting-edge security goals like future secrecy (Lyu, Gharakheili & Sivaraman, 2022). Automatically, a secure and sound communication protocol should deliver a neck and neck of security equivalent to interpersonal communication in a safe area. Both in the room overhear the communication, both recognize who spoke and how frequently words have been spoken, and no one outdoor the apartment can either say towards the room or listen to the conversation inside, and the door of the apartment is unlocked only for asked peoples (Sun et al., 2022; Zhang, He & He, 2023).

### Notations and assumptions

**Figure 2:**
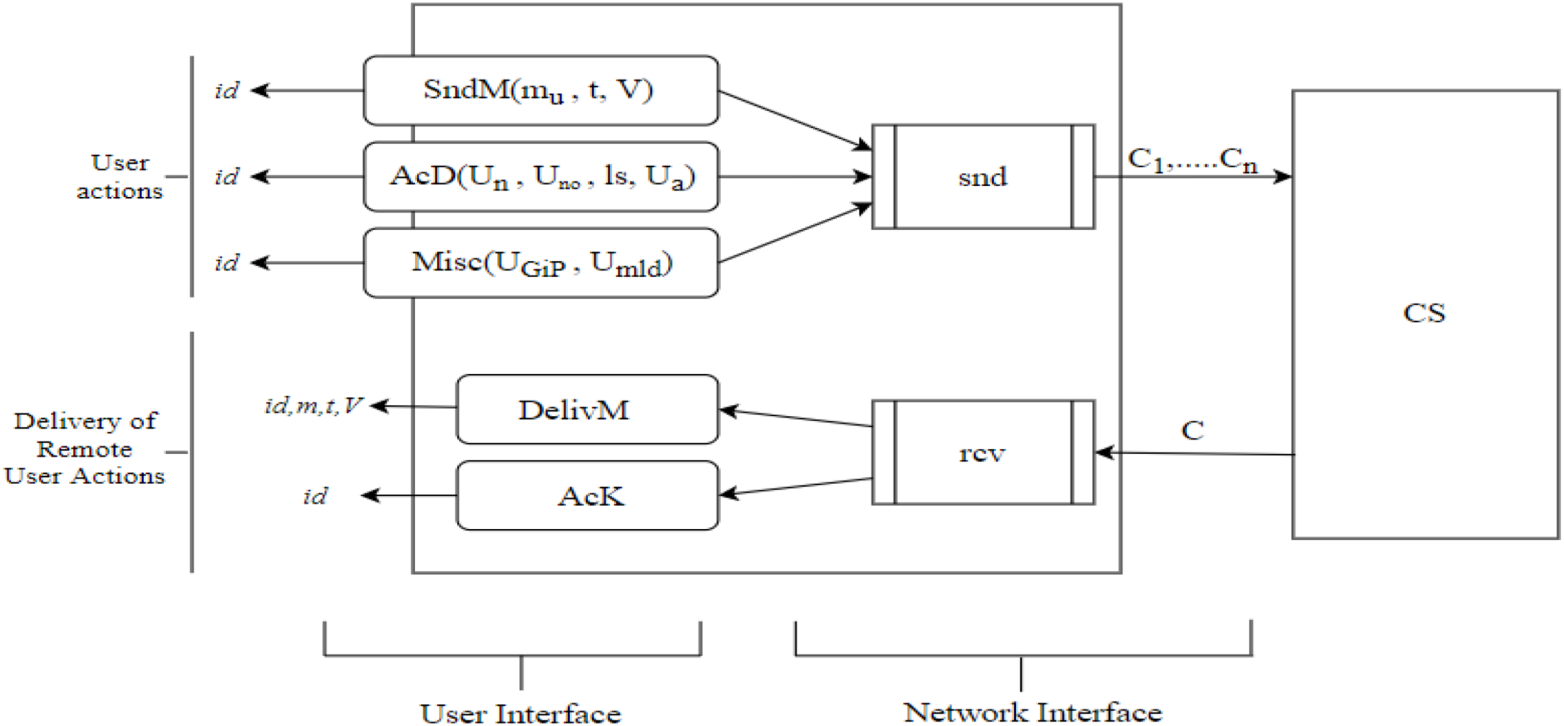
Summary of the syntax of IM protocols showing the cooperating user’s interfaces and the boundaries of the application to the network.

In reality, the IM protocols are centralized. All communicated messages are communicated through a centralized server that receives messages from the individual senders, stores them, and forwards them as soon as the receivers are online. That is why the protocols are performed in an asynchronous atmosphere in which only the server remains online, as shown in Fig. 2. The algorithms first handle the message and then the result is delivered to the end-user. The notions and terms used for cloud-based security model are shown in Table 1.

**Table 1:** Notation guide used in the cloud-based security model.

Notation	Description
*SndM*	Message sending algorithm
*AcD*	Account details algorithm
*Misc*	Another info-based algorithm
*DelivM*	Message delivery
*AcK*	Acknowledgment algorithm
*C*	Encrypted text
*V*	Vector of encrypted texts
*m*	Message
*ID_u_*	Unique user identifier
*id*	Unique reference string
*m_t_*	Message text
*t*	Time
*C_n_*	User’s account name or title
*U_no_*	User’s contact number
*ls*	Last seen
*U_a_*	User about (bio)
*U_GiC_*	User groups in common
*U_mld_*	User media, links, documents
*snd*	Sending algorithm
*rsv*	Receiving algorithm

Let us define a message as a tuple.

$m = (I{D_u},\; {m_t},\; t),\; I{D_u} \subseteq \left\{ {\left( {{U_n},{U_{no}},ls,\; {U_a}} \right) \cup \left( {{U_{GiC}},\; {U_{mld}}} \right)} \right\}\; \subseteq {\rm \mu }.$

Here is the finite set of user protocols. $I{D_u}$ is the set of User IDs containing the username, user contact number, last seen if it is visible to all, and user bio if its setting is set to be public. If there is already communication done, then we can also have it ${U_{GiC}},\; {U_{mld}}$.

The user is uniquely referenced on a central server and contains and $Misc$. We have donated encrypted communication as ${C_1},{C_2}, \ldots ,{C_n} \in \; \mu$. Every user on the communication network maintains the long-term secrets of starting communication with other users and session states. Messages delivered to an end-user are not saved in the state of a session. By differentiating the delivery of the messages and receiving, we need to highlight that the algorithms first handle the message received and then the result is shown to the end-user.

### Asymmetric key encryption scheme

We have proposed an asymmetric-key encryption scheme. The scheme is used for encryption purposes to hold privacy with the generated session key on Simple Matrix for the security of the message. The resulting representations are used for instant messaging (IM) to show the scheme using asymmetric key encryption, as shown in Table 2.

**Table 2:** Terms used for the asymmetric key encryption scheme.

Notation	Description
Variable i	$\frac{q}{2}$
Public key	P = G _°_ M _°_ H: → in F
Private key	Matrices Y, Z and two linear maps G, H in F
Variable g	$\sqrt{n}$
Number of finite field elements	e
F and Central map	M in F
Quadratic polynomials in number	q in F
Three matrices with the size of $g \times g$	X, Y, and Z in F
Plaintext and Ciphertext	$j \in F^{n} \;and\; u \in F^{m}$
Two linear maps	G and H in F

**Figure 3:**
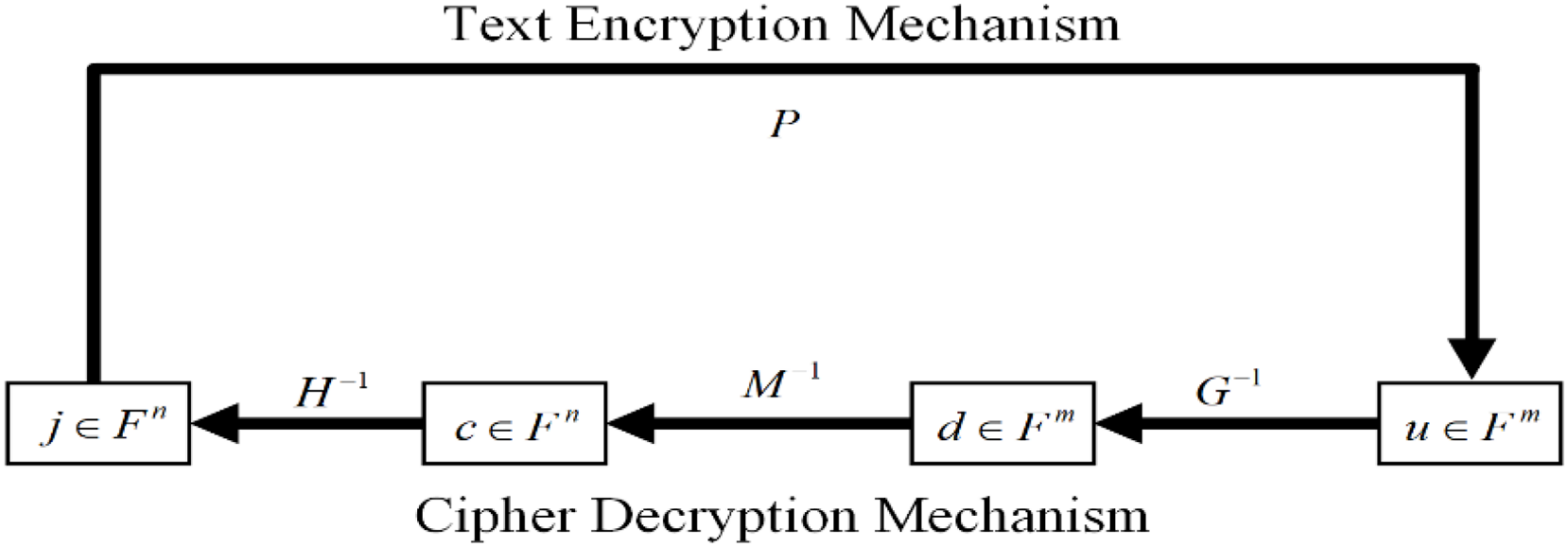
Encryption and decryption mechanism.

We have summarized the detailed information on the encryption scheme, which we have outlined in Fig. 2. The encryption process is unpretentious. To encrypt a message, the public P must compute $u = P(j) \in {F^m}$. This process is done by using the polynomial evaluations. Since the security of the encryption scheme is based on solving quadratic equations to decrypt a cipher text you ∈. We have summarized the encryption and decryption process in Fig. 3. We can generate two linear maps, G and H in F, and the private key with matrices Y and Z. After this process, the private keys are used to calculate the public key in the form of an F. Therefore, it is compulsory to complete the above-mentioned three steps to complete the process.

First, it $d = {G^{( - 1)}}(u)$ is computed as shown in Eq. (1).

(1) $$d = {G}^{\prime - 1}u.$$

${G}^{\prime}$is a matrix of q × q. Secondly, $c({c_1},{c_2},...{c_n}) = {M^{( - I)}}(d)$ it is required to be computed. We suppose that and denote the matrices in the following forms.

$E^\prime{q_1} = \left[ {\matrix{ {{d_1}} & {{d_2}} & {...} & {{d_g}} \cr {{d_{g + 1}}} & {{d_{g + 2}}} & {...} & {{d_{2g}}} \cr {{d_{2g + 1}}} & {{d_{2g + 2}}} & {...} & {{d_{3g}}} \cr \ldots & \ldots & \ldots & \ldots \cr {{d_{(g - 1)g + 1}}} & {{d_{(g - 1)g + 2}}} & \ldots & {{d_n}} \cr } } \right] E^\prime{q_2} = \left[ {\matrix{ {{d_{i + 1}}} & {{d_{i + 2}}} & {...} & {{d_{i + g}}} \cr {{d_{i + g + 1}}} & {{d_{i + g + 2}}} & \ldots & {{d_{i + 2g}}} \cr {d{i_{i + 2g + 1}}} & {{d_{i + 2g + 2}}} & \ldots & {{d_{i + 3g}}} \cr \ldots & \ldots & \ldots & \ldots \cr {{d_{i + (g - 1)g + 1}}} & {{d_{i + (g - 1)g + 2}}} & \ldots & {{d_m}} \cr } } \right].$

We have to calculate the inverse of ${{E}^{\prime}_{q1}}$, *i.e*., if invertible. We have computed $j = {H^{ - 1}}\left( c \right)$ in the following form as shown in Eq. (2) by constructing the variables ${c_1},{c_2},{\rm }...{c_i}$. If none of $E{'_{q1}},E{'_{q2}}$ and $X'$ is invertible then decryption process fails. We have constructed i linear equations on i variables ${c_1},{c_2},{\rm }...{c_i}$ based on $X{'^{ - 1}}{\rm \times\, }E{'_{{q_1}}} - Y = 0$, and $X{'^{ - 1}}{\rm \times\, }E{'_{q2}} - Y = 0$. We have unraveled the equations on variables ${c_1},{c_2},{\rm }...{c_i}$ if none of $E{'_{q1}}$ or $E{'_{q2}}$ is invertible, but $X' = X\left( c \right)$ is invertible, *i.e*., $X{'^{ - 1}}$. We have constructed i linear equations on i variables ${c_1},{c_2},{\rm }...{c_i}$. based on $Z{\rm \times\, }E{'_{q2}}^{ - 1}{\rm \times\, }E{'_{q1}} - Y = 0$. It is necessary to calculi the variables ${c_1},{c_2},{\rm }...{c_i}$ if ${{E}^{\prime}_{q1}}$ is not invertible, but ${{E}^{\prime}_{q2}}$ is invertible, calculate $E{'_{{q_2}}}$, *i.e*., ${{E}^{\prime}_{q2}}^{ - 1}$. We have constructed n linear equations on n variables ${c_1},{c_2},{\rm }...{c_i}$ based on $Y{\rm \times\, }E{'_{q1}}^{ - 1}{\rm \times\, }E{'_{q2}} - Z = 0$.

(2) $$j{\rm } = T{'^{ - 1}}c.$$

${T}^{\prime}$ is a matrix of i × i. The asymmetric-key encryption scheme can retain confidentiality after that, the plaintext j has been calculated.

### Signature generation scheme for public key

Table 3 represents the public-key signature generation scheme with the given notations.

**Table 3:** Terms and notions used by signature generation scheme for public key.

Notation	Description
Number of finite field elements	F
Affine transformation matrix 1	*K_G_* in F
Affine transformation matrix 2	K_H_ in F
The matrix for central map transformation	M in F
The signature’s size	J
Vector *x* in *K* with the signature	$z\left(z_{0}, z_{1}, \ldots,Z_{j-1}\right)$
The size of the communication digest	I
Vector x in F with the form of message	$x\left(x_{0}, x_{1},\ldots,x_{j-1}\right)$
Public key	$\bar {P}=K_{G}\;\circ\; M\;\circ\;K_{H}\;in \;F$
Private key	Three transformations K_G_, M and K_H_ in F

We have summarized the detailed information on the signature scheme. Three transformations ${K_G}$, M and ${K_H}$ in F are used to generate the private key. The private keys are used to calculate public key, *i.e*., $\overline P = {K_G} \circ M \circ {K_H}\,in\,F$. The quadratic equations in F are used in the signature scheme for the security of data. In order to sign an encrypt message $x\left( {{x_0},{x_1},...,{x_{j - 1}}} \right) \subset F$, it is required to solve the Eq. (3).

(3) $$M \circ {K_h}\left( {{z_0},{z_1},...,{z_{j - 1}}} \right) = {k_1}^{ - 1}\left( {{x_0},{x_1},...,{x_{j - 1}}} \right).$$

We require to calculate the hash value of the message to solve (Eq. (3)) by using a SHA-256 based hash function.

(4) $$x^\prime =hash (x).$$

We have to calculate the affine transformation matrix LS, secondly.

(5) $$\overline x = {k_1}^{ - 1}\left( {x{'_0},x{'_1},...,x{'_{j - 1}}} \right).$$

Third, the central map transformation M is calculated using Eq. (5).

(6) $$\overline x = {M^{ - 1}}\left( {x{'_0},x{'_1},...,x{'_{i - 1}}} \right).$$

Fourth, affine transformation matrix ${K_H}$ is calculated based on the calculation outcome of Eq. (6).

(7) $$z = {K_H}^{ - 1}\left( {\overline {{z_0}} ,\overline {{z_1}} ,...,\overline {{z_{j - 1}}} } \right).$$

The signature $z\left( {{z_0},{z_1},...,{z_{j - 1}}} \right) \subset F$, we have to calculate the Eq. (8) to verify the process of the signature verification, which is simple. Finally, we generate the signature z.

(8) $${x}^{\prime \prime}({x_0}^{\prime \prime },{x_1}^{\prime \prime },.....,{x_{i - 1}}^{\prime \prime }) = \overline M \left( {{z_0},{z_1},...,{z_{j - 1}}} \right).$$

If ${x}^{\prime \prime}$ = ${x}^{\prime}$, then the signature is acceptable by comparing ${x}^{\prime \prime}$ with the hash value of the original message ${x}^{\prime}$. In other case the case is rejected.

### Secure communication system

**Figure 4:**
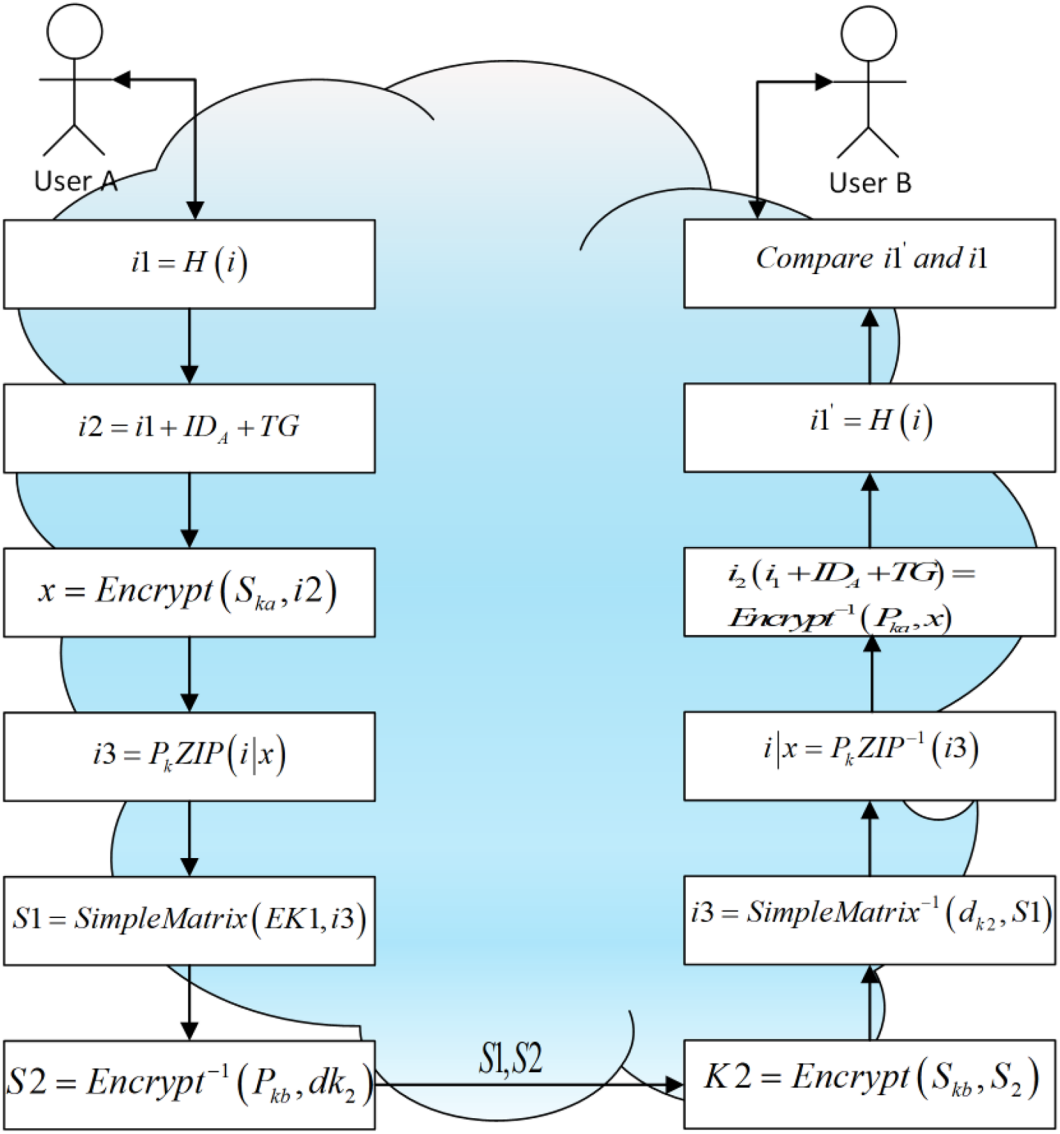
A secure communication system between two users in the cloud environment.

The complete communication process is shown in Fig. 4. The communication among the three main users must be protected by security. We use ‘A’ and ‘B’ to denote the user of the cloud client and the user of the cloud service platform respectively (Lakshmi et al., 2022). To do this, the entities must communicate in a manner that is impervious to eavesdropping or interception. When two entities communicate and do not want a third party to listen in, they use secure communication.

Sender A:

The user ‘A’ needs a safe way to share the information i to the user ‘B’.

The user ‘A’ generates the hash value of i based on SHA-256 by using a hash function with a 256-bit digest, *i.e*., i1 = H(i).

${i_2} = {i_1} + I{D_A} + TG$ is used, where TG is a timestamp with 128 bit long and $I{D_A}$ is the user A’s ID with 32 bit long. The ID of the user ‘A’ and timestamp are appended to ${i_1}$.

The sender ‘A’ generates ${P_{ka}}$ and ${S_{ka}}$ which is the public key and private key of the signature scheme.

The public key of the sender ‘A’ is public, *i.e*., ${P_{ka}}$ and private key ${S_{ka}}$ is held in reserve in a private way.

The sender ‘A’ uses ${S_{ka}}$ gets the signature x which is 344 bits by signing i2 and it is based on signature scheme, *i.e*., x = Encrypt ( ${S_{ka}}$, i2).

The user ‘A’ compress (i|x) based on ${P_k}$ ZIP, *i.e*., i3 = ${P_k}$ ZIP(i|x).

‘A’ generates $E{k_1}$ and $d{k_2}$ for encryption and a decryption key of the scheme.

‘A’ encrypts S1 = SimpleMatrix ( $E{k_1}$, i3) by using the encryption key $E{k_1}$ to encrypt i3 based on the encryption scheme.

‘A’ uses S2 = Encrypt−1 ( ${P_{kb}}$, $d{k_2}$) to encrypt the decryption key $d{k_2}$ by using user B’s public key ${P_{kb}}$ based on the encryption scheme.

‘A’ sends S1 and S2 to user ‘B’ in a public way.

Receiver B:

The receiver ‘B’ decrypts S2 and gets the decryption key based on the encryption scheme $d{k_2}$, *i.e*., $d{k_2}$ = Encrypt ( ${S_{kb}}$, S2) by using the private key of the signature scheme ${S_{kb}}$.

The receiver ‘B’ calculates i3 = $SimpleMatri{x^{ - 1}}$ ( $d{k_2}$, S1) by using $d{k_2}$ to decrypt S1 based on the encryption scheme.

The receiver ‘B’ calculates i|x = ${P_k}$ ZIP − 1 (i3) by decompressing i3 based on ${P_k}$ ZIP.

The receiver ‘B’ calculates i2 (i1 + $I{D_A}$ + TG) = $Encryp{t^{ - 1}}$ ( ${P_{ka}}$, x) by using user A’s public key ${P_{ka}}$ to verify the signature s.

The receiver ‘B’ calculates $SimpleMatri{x^{ - 1}}$ = H (i) by using a SHA-256 hash function to generate the hash value of m.

The receiver ‘B’ compares $i{1}^{\prime}$ and i1. If it has been tampered the values are different otherwise, i is original.

### Traffic classification methodology

**Figure 5:**
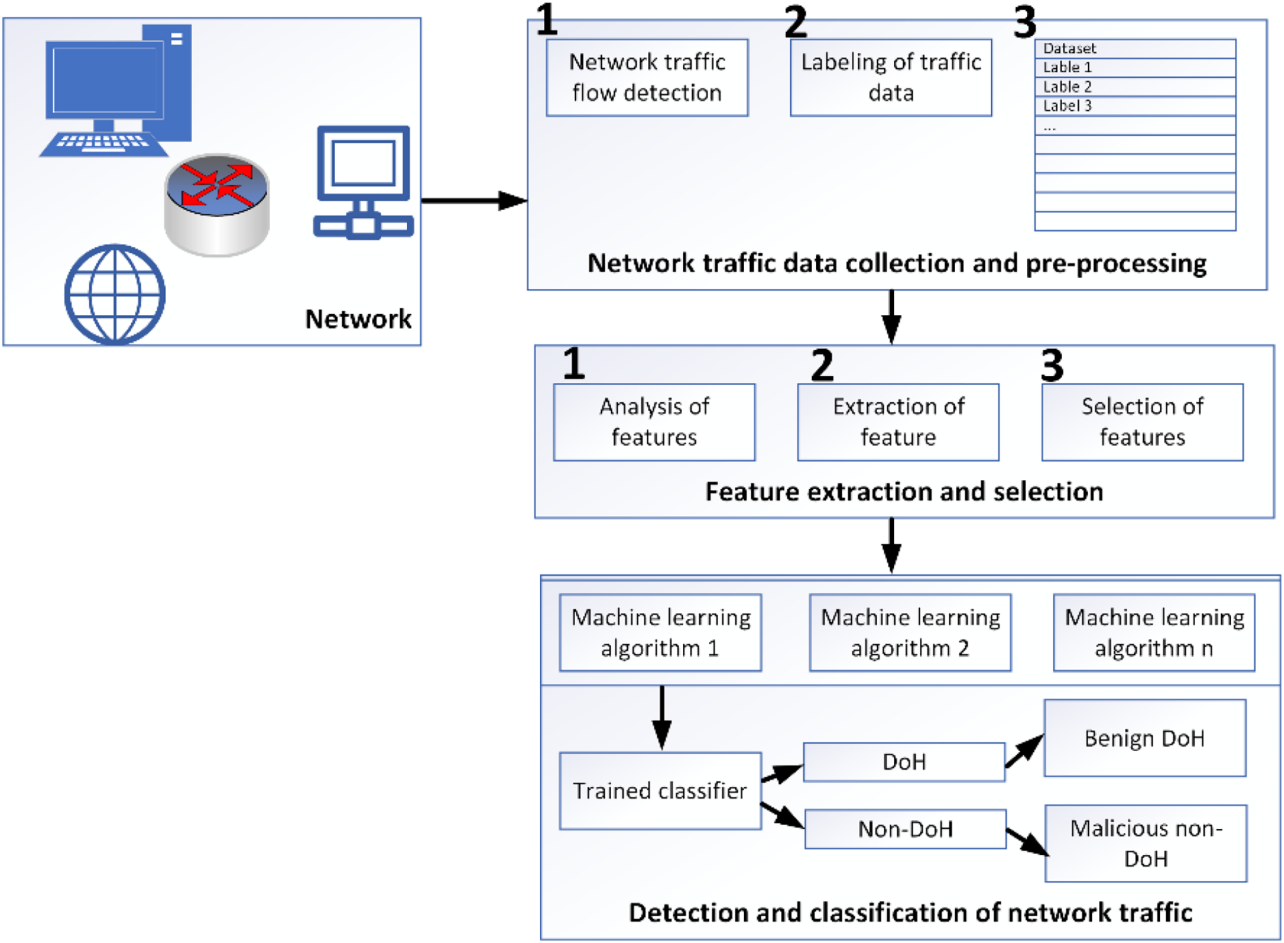
Methodological framework for data capturing, analyzing and classifying (MontazeriShatoori et al., 2020).

Various programs have recorded HTTPS traffic for use in the training dataset. The tuples include protocol detail, source port, destination port, source IP, and destination IP. The pre-processing data module identifies every collected data flow from the encrypted network traffic. The dataset is labeled according to the IP address of the flow’s final destination because the protocol (TCP) and destination port (443) for all flows are the same. In addition, techniques utilized to generate DoH flows set them apart. Simulated DoH flows are labeled as benign, whereas DoH tunnel-captured flows are labeled as malevolent (Liu et al., 2021). When it comes to the DoH protocol, malevolent actors may utilize it to build covert channels in several ways. We name this DoH tunneling network traffic “malicious,” as shown in Fig. 5.

To facilitate reproducibility and bolster scientific rigor, additional specifics are warranted regarding model evaluation protocols and implementation details. The selection of accuracy, AUC, confusion matrices and other metrics presented herein stem from recommended best practices for multi-class traffic classification tasks. Furthermore, while baseline default parameters suffice initially, model optimization *via* tuning of key hyper parameters (*e.g*. kernel type, regularization, ensembling parameters *etc*.) can yield substantial improvements. Therefore, the model training process undertaken involves systematic grid search over viable hyper parameter ranges for each algorithm. The optimal configurations obtained after sweeping through hundreds of combination yielding the highest cross-validation performance are finally locked in. Such iterative tuning of model knobs to find the ideal operating point that generalizes well allows us to maximize effectiveness. By elucidating factors behind metric choices for model selection, specifying tuning heuristics adopted, the research process is rendered more transparent. Augmenting these fine-grained specifics bolsters methodological rigor and aids reproducibility by qualitatively articulating a structured approach to optimizing ML pipeline performance through evidence-driven customization of learning schemes presented.

### Dataset detail and pre-processing

Among the earliest and most susceptible network protocols, the DNS has repeatedly exploited several security flaws over the years. In cybersecurity, DNS abuse has long been a major source of worry. Although attackers utilize advanced attack tactics to steal data on the fly, ensuring security and privacy for DNS queries and answers is still a difficult challenge to do (Gopi et al., 2022). IETF established DNS over HTTPS (DoH) in RFC8484 to address some DNS privacy and data manipulation issues. DoH encrypts DNS queries and sends them over an encrypted covert channel/tunnel, ensuring that data is not harmed in transit. However, the lack of a representative dataset makes evaluating the methods for capturing DoH traffic in a network architecture difficult. DoH traffic through covert channels and tunnels that are studied, tested, and evaluated using a systematic manner proposed in this study. In order to identify and analyze DoH traffic using a time-series classifier, this research aims to install DoH inside an application and capture both benign and malicious DoH traffic. Data were collected as previously described in Abid et al. (2023).

Using five different browsers and tools and four servers, the final dataset comprises DoH protocol implementation in an application that captures benign-DoH, malicious-DoH, and non-DoH traffic. On the first tier of the two-layered technique described, DoH communication is classified as either benign or malicious depending on whether it comes from a DoH device. Search engines like Google Chrome employ many different methods to collect traffic, such as DNSCat2, DNSCat3, and Iodine, while servers like Cloudflare and Google DNS reply to DoH requests using AdGuard and Cloudflare respectively. Initially, the dataset is pre-processed by encoding the source and destination IP addresses and time stamp values using an ordinal encoder. The NA values are dropped, as shown in Algorithm 1.

**Algorithm 1:** Data encoding algorithm.

**Input:** Raw data in the form of a table or data frame
**Output:** Encoded data
Retains only non-null values and drops all NA-values
Reshapes the data for encoding by imputing it
Encode data using an ordinal encoder
Assign back encoded values to non-null values in the original data
Iterate through each column in the data as
**for** columns **in** category columns:
encode (data Frame[columns])
**return** encoded data

The used ML algorithms are described as:

DT Minimize entropy $H(T)$ to construct a tree $T$: $T:T = \arg \min {\lim _T}H(T)$ Naive Bayes Apply Bayes rule, assuming conditional independence between features as shown in Eqs. (9) and (10).

(9) $$P(y|x) = \displaystyle{{P(y)P(x|y)} \over {P(x)}}.$$

(10) $$P(x|y) = \prod\limits_{i = 1}^n P ({x_i}|y).$$

K-nearest neighbors classify by majority vote of the $K$ nearest samples in feature space $\hat y = {\rm mode}{y_i}:i \in {N_K}(x)$ neural network learn feature transformations $f()$ and classification $g()$ by optimizing a loss function over parameters $\theta$: $\mathop {\min }\limits_\theta L(y,g(f(x;\theta )))$ QDA Assume Gaussian distributions per class and find boundaries as shown in Eq. (11):

(11) $${\delta _k}(x) = {x^T}\Sigma _k^{ - 1}x + {x^T}\Sigma _k^{ - 1}{\mu _k} - \displaystyle{1 \over 2}\mu _k^T\Sigma _k^{ - 1}{\mu _k} + \ln P(y = k).$$

RF aggregate predictions from $N$ randomized DTs as shown in Eq. (12):

(12) $$\hat y = {\rm mode}{T_1}(x),{T_2}(x),...,{T_N}(x).$$

SVM (RBF kernel) maximize margin between classes with nonlinear decision boundary as shown in Eq. (13):

(13) $$f(x) = {\rm Maximize}\left( {\sum\limits_{i = 1}^N {{\alpha _i}} {y_i}K({x_i},x) + b} \right)$$

where $K(x,{x}^{\prime}) = \exp ( - \gamma |x - {x}^{\prime}{|^2})$ is the RBF kernel.

## Results and discussion

**Figure 6:**
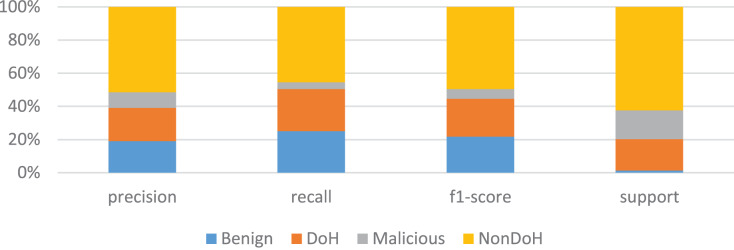
KNN model training results in the form of precision, recall, F1-score and support.

The K-nearest neighbors with the value of k is four is used; hence, the total number of classes is four. The dataset is divided into two parts, *i.e*., training and testing parts. The training part consists of 67% of the data, and 33% of data is carried for the testing dataset. The overall accuracy of the model is 75%, which is not very good, but it can be increased during the full experiments. The results for NonDoH are very good, but for DoH, benign and malicious are very promising. The precision is good for benign, while recall and F1-score are better for DoH, as shown in Table 4 and Fig. 6.

**Table 4:** Statistical measures of the K-NN with K = 4.

	Precision	Recall	F1-Score	Support
Benign	0.37	0.55	0.44	6,464
DoH	0.39	0.56	0.46	88,639
Malicious	0.18	0.09	0.12	82,555
NonDoH	1	1	1	293,716
Macro avg	0.48	0.55	0.50	471,374
Weighted avg	0.73	0.75	0.73	471,374

A comparative analysis between this article and some of the key references on malicious DNS traffic detection using ML techniques is summarized in Table 5. As shown in the table, while existing literature has explored related problems, this article makes several key contributions in terms of the dataset diversity, proposed methodology, and rigorous ML pipeline evaluation as well as classification performance. The key differentiation of this two-layered classification approach leveraging time series features is highlighted across various comparative aspects against prior art.

**Table 5:** Comparison between key contributions of our work and existing literature on malicious DNS traffic detection using machine learning.

Comparison aspects	Our work	Vries (2021)	Singh & Roy (2020)	AlQaralleh et al. (2022)	Tu et al. (2021b)
Problem addressed	Detection & classification of malicious DoH traffic using ML	Detection of DNS tunnels *via* ML	Detecting malicious DoH traffic by ML	Identifying malicious DNS tunnels from DoH traffic by ML	Review of ML for security of DNS including malicious query detection
Dataset	Custom dataset with diverse benign, malicious DoH & non-DoH traffic	No dataset details provided	No dataset details provided	No dataset details provided	Various standard datasets referenced
Learning approaches	SVC, QDA, AdaBoost (high accuracy)	Supervised & unsupervised ML compared	RF, KNN, GB evaluated	Hierarchical ML classification	Survey of different ML techniques
Traffic classification	Uniquely two-layered approach	Single layer tunnel detection	Labeling based on IP addresses	Focus only on tunnel identification	NA
Key attributes	Custom data collection strategy and features, advanced ML evals, two-layer methodology	Compares supervised & unsupervised ML	Basic ML models evaluated	Hierarchical classification approach	Broad review of techniques
Outcomes	Systematic evaluation and high accuracy multi-class results	Methodology comparison, no accuracy reports	No performance results given	No accuracy results provided	Review of landscape

**Figure 7:**
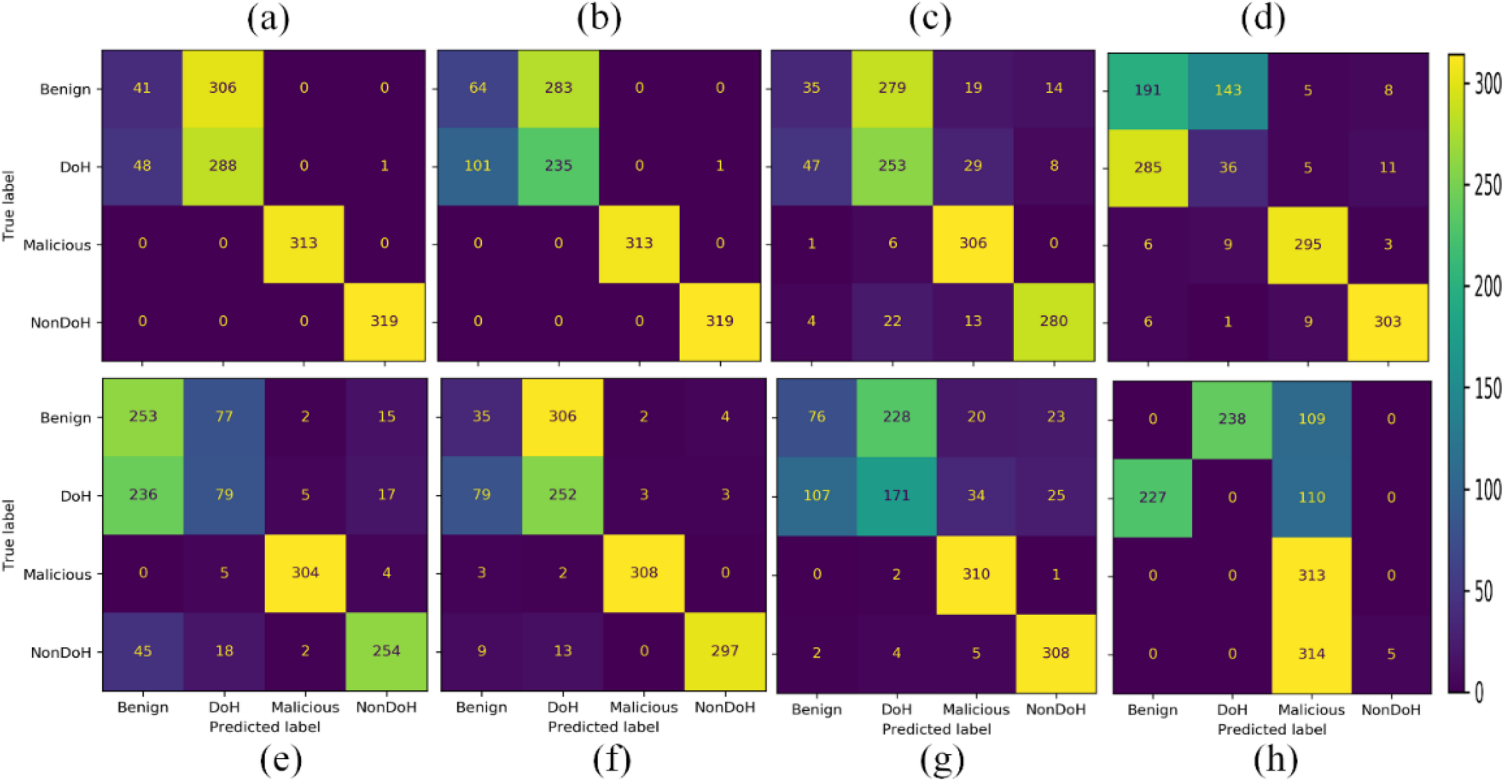
Confusion matrices of the classifiers as (A) AdaBoost (B) decision tree (C) naïve Bayes (D) nearest neighbors (E) neural network (F) quadratic discriminant analysis (G) random forest (H) support vector classification with RBF.

Eight different ML models are trained in four classes. The classes predicted by some models are very distant, while others got false positive and false-negative results. The overall results in a confusion matrix are shown in Fig. 7. The Ada Boost, DT classified malicious, and non-DoH without any confusion. While other models also classified these classes with much better accuracy, except for SVC-RBF. The other classes by other models, *i.e*., DT, naïve Bayes (NB), nearest neighbors, neural network, QDA, RF, and SVC-RBF, have some problems.

**Figure 8:**
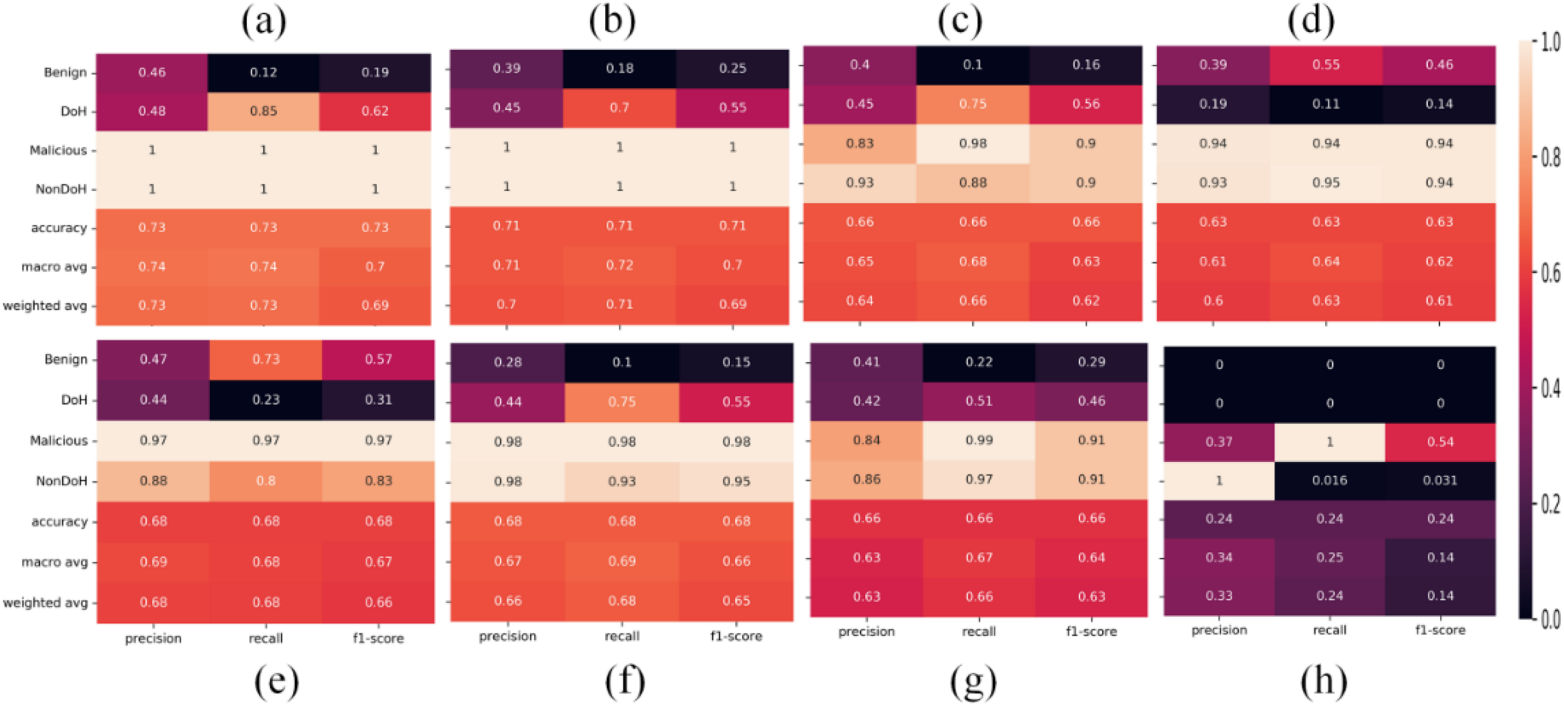
Statistical summaries of the classifiers as (A) AdaBoost (B) decision tree (C) naïve Bayes (D) nearest neighbors (E) neural network (F) quadratic discriminant analysis (G) random forest (H) support vector classification with RBF.

Statistical measures summaries like per class accuracy, overall accuracy, macro average accuracy, and weighted accuracy obtained from ML classifiers, *i.e*., AdaBoost, DT, NB, nearest neighbors, neural network, QDA, RF, SVC-RBF are shown in Fig. 8.

**Figure 9:**
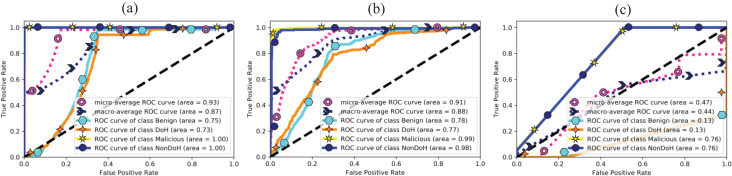
AUC for (A) AdaBoost, (B) quadratic discriminant analysis, and (C) support vector classification with RBF.

The area under curve obtained from ML classifiers, *i.e*., AdaBoost, QDA, and SVC-RBF, as shown in Fig. 9. The SVC-RBF model classified Malicious as 76% and non-DoH as 76%, benign got 13%, and DoH class got 13% accuracy. The macro accuracy is 44%, and the micro average accuracy is 47%. The QDA model classified malicious as 99% and non-DoH as 98%, benign got 78%, and DoH class got 77% accuracy. The macro accuracy is 88%, and the micro average accuracy is 91%. The AdaBoost model correctly classified malicious and Non-DoH while benign got 75% and DoH class got 73% accuracy. The macro accuracy is 87%, and the micro average accuracy is 93%.

**Figure 10:**
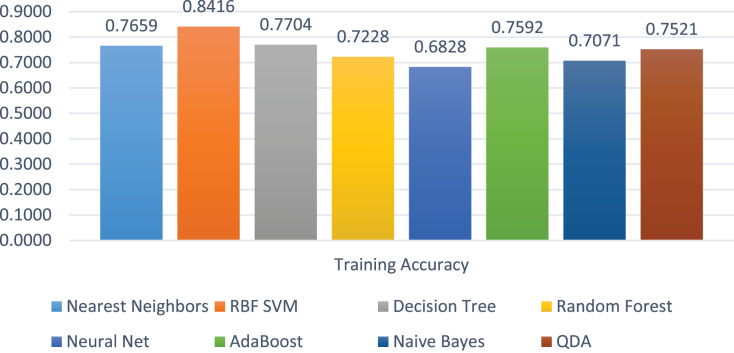
Models’ training accuracies in all the four classes.

The training accuracies of all the models are shown in Fig. 10. The training accuracy of SVC-RBF is the highest among all other models, *i.e*., 84%. The comparatively other models performed less while training them. The range of the training score is 68% to 84% on four class labels.

**Figure 11:**
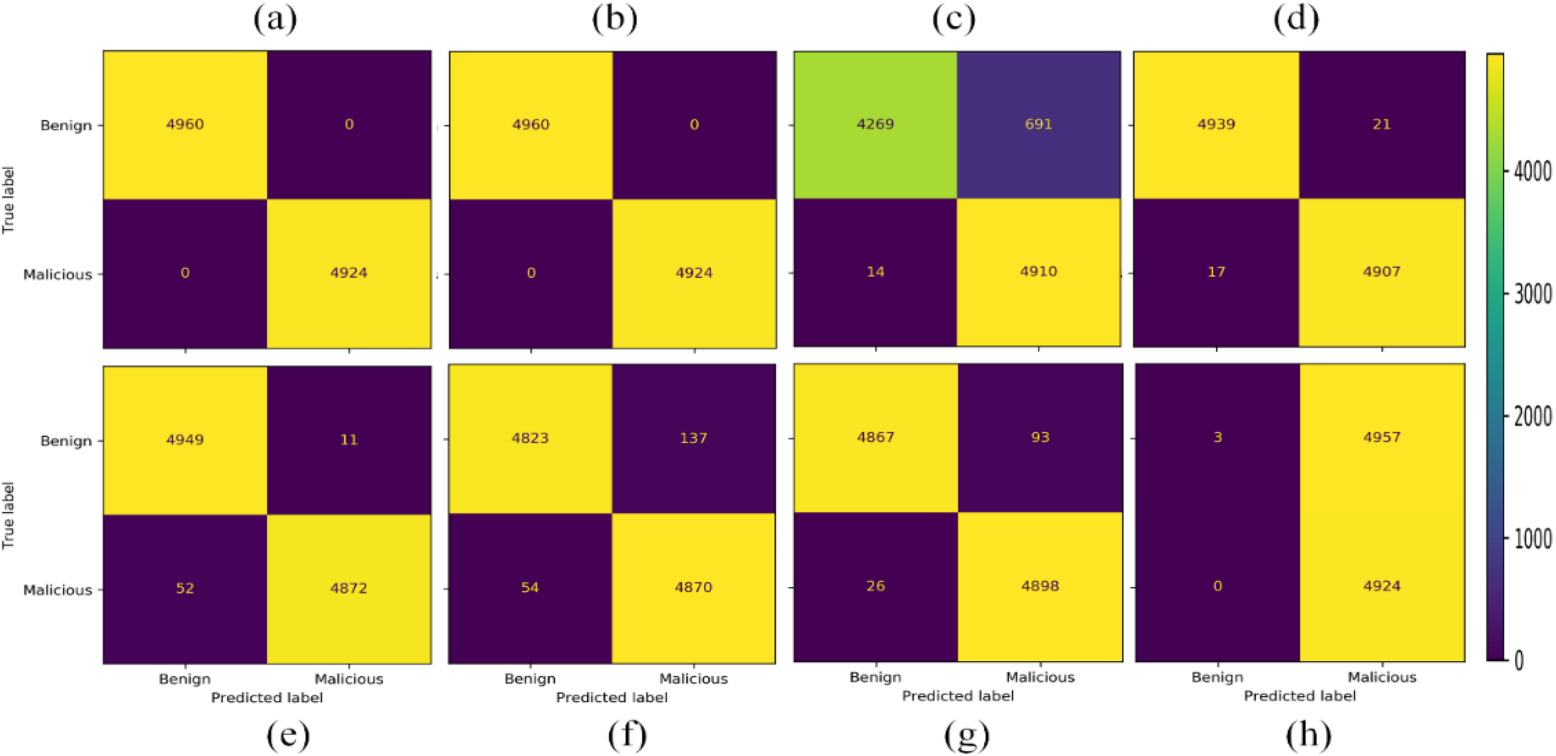
Confusion matrices with 15,000 samples each class (A) AdaBoost (B) decision tree (C) naïve Bayes (D) nearest neighbors (E) neural network (F) QDA (G) random forest (H) SVC-RBF.

The next experiment is conducted by selecting two class labels from the dataset, *i.e*., Benign and Malicious. In this scenario SVC-RBF model failed as it has classified almost all the data as the malicious class label. Therefore, the SVC-RBF model is not suitable for the classification of this dataset. NB model performed better as compared to SVC-RBF. The other models like nearest neighbors, neural network, QDA, and RF performed moderately. The best-performing model for this given problem in the given scenario is AdaBoost and DT, as shown in Fig. 11.

**Figure 12:**
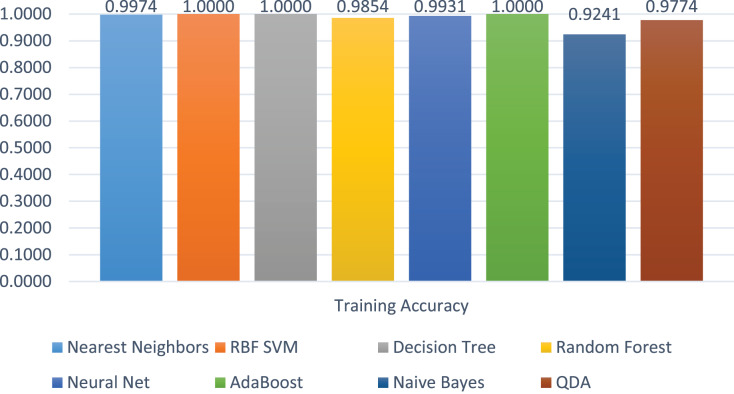
Models’ training accuracies on benign and malicious classes.

The training accuracies of all the models are shown in Fig. 12. The training accuracy of SVC-RBF, DT, and AdaBoost is higher among all other models. The comparatively other models performed less, *e.g*., RF, Neural Network, and NB. The range of the training score is 92% to correct classification on 2 class labels.

We explore an important problem regarding classification of encrypted DNS traffic using ML, the specific research questions and knowledge gaps being addressed could be more clearly positioned. The authors should outline the precise real-world issues and limitations in existing methodologies that this work aims to tackle. For example, the introduction could highlight open questions around rigorously benchmarking complex ML algorithms for multi-class encrypted traffic analysis, and the lack of diversity in current DNS tunneling datasets. It can cite the dependency on standard corpora and single tunneling tools in prior approaches as an inherent limitation. Building on this problem framing, the novel contributions proposed—including the two-layered methodology, focus on time-series characterizations, and data collection strategy spanning browsers and tunneling tools—can be presented as targeted efforts to fill these gaps. By first discussing the specific open research questions on applying ML to DNS security, assessing alternatives, and articulating limitations therein, this work can concretely situate how their technical approach and results advance knowledge over documents in literature. The comparisons should emphasize dimensions such as model sophistication, dataset diversity, classification granularity *etc*. as differentiators to strengthen claims around addressed knowledge gaps. Enhancing this contextual framing of research issues, current shortcomings, and targeted improvements will help accentuate the significance of innovations introduced by the authors in the ML pipeline for encrypted DNS traffic analytics.

## Conclusions

Computer networks have become simple targets for cyber-attacks in the ever-changing internet services. DNS assaults have been greatly reduced because of HTTPS. DoH is used to help protect against Man in the Middle attacks by fighting eavesdropping and DNS data tampering during DNS communication. The attacker utilizes advanced attack tactics to steal data for DNS queries, and answers are still a difficult challenge. The network traffic data detection and categorization using ML methods are done using different classifiers. The four classes-based classifications and two classes-based classifications are done in different experiments. It is found that for the four classes, the SVC-RBF model achieved 76% accuracy. The QDA model achieved 99% accuracy. The AdaBoost model correctly classified malicious and non-DoH classes. The 2-class scenario found that the training accuracy of SVC-RBF, DT, and AdaBoost is higher among all other models.

This study aimed to investigate the application of ML techniques for detection and classification of DoH network traffic. Specifically, it sought to evaluate different models for identifying and distinguishing between benign, malicious and non-DoH communications within an encrypted traffic dataset.

The results demonstrate that the two-layered classification approach is highly effective at analyzing DoH traffic in depth. At layer one, the support vector classifier with RBF kernel achieved 76% accuracy in differentiating between malicious and non-DoH traffic. Meanwhile, at layer two, the QDA model attained classification rates of 99% and 98% for malicious traffic and non-DoH traffic respectively. The AdaBoost ensemble classifier also performed well, with accuracies of 75% and 73% for benign and DoH classes.

Notably, the time-series feature engineering based on packet clump representations enhanced encrypted traffic learnability. This validates the hypothesis that new learning representations tailored for HTTPS data payloads can improve detection quality.

In conclusion, the findings strongly support the research question by showing ML provides a viable solution for DoH network analysis. Classification performance often exceeded 90% for models trained on the custom dataset. This contributes significantly to knowledge by demonstrating ML is practical for encrypted DNS traffic understanding. Going forward, the two-layer framework and proposed feature set warrant further exploration on more extensive real-world DoH traffic corpora. With refinement, such techniques show promise for bolstering security and surveillance of encrypted network protocols.

As we look towards the future, it is clear that our work must continue to evolve alongside the complexities and variety of cyber threats that are also increasing. Despite the promising results that ML methods have demonstrated in the realm of network traffic data detection and categorization, the challenges posed by advanced attack tactics cannot be underestimated. Therefore, our next steps will involve several key areas of focus. We aim to improve multi-class classification by refining the SVC-RBF and QDA models that have shown good initial results. Our goal is to explore a wider range of ML and deep learning algorithms for this purpose, with the intent to achieve even higher accuracy levels in multi-class classification of network traffic data. In the context of binary classification, the superior training accuracy of the SVC-RBF, DT, and AdaBoost models in a 2-class scenario has pointed us towards a future validation of these models on different datasets. We will concentrate on enhancing the detection rate of malicious traffic while simultaneously minimizing both false positives and negatives. With the prevalence of advanced and dynamic attack tactics used by cyber criminals, it is paramount to develop ML models that are capable of learning and adapting to these tactics over time. Such an approach will help us maintain an edge over cyber threats, and ensure robust security for DNS queries. Although DNS over HTTPS (DoH) has significantly reduced the frequency of DNS attacks, guaranteeing the security and privacy of DNS queries and responses remains a formidable challenge. Therefore, our future work will also focus on devising additional security measures to enhance the effectiveness of DoH. Recognizing the potential advantages of cloud technology, we plan to investigate cloud-based solutions for managing network traffic data. The scalability and distributed nature of the cloud could be harnessed to handle large-scale data more efficiently.

## Supplemental Information

10.7717/peerj-cs.2027/supp-1 Supplemental Information 1 Dataset for stateless attack as heavy and All Processed. A statistical characteristics classifier divides the collected traffic into two categories: DoH and non-DoH. DoH traffic is classified as either benign or malicious at the second layer using a time-series classifier. Non-DoH: Accessing a website using the HTTPS protocol generates traffic designated as non-DoH. Many Alexa domain websites are visited to ensure the dataset is well-balanced. ‘Benign-DoH’ is non-malicious DoH traffic created using the same method as in ‘non-DoH’ by utilizing the Mozilla Firefox and Google Chrome web browsers’ This is known as malicious DoH traffic and is generated by DNS tunneling software such as dns2tcp, DNSCat2, and Iodine.

10.7717/peerj-cs.2027/supp-2 Supplemental Information 2 Python Code for Data Pre-Processing.

10.7717/peerj-cs.2027/supp-3 Supplemental Information 3 Python Code for Multiple Models' Training and Testing.

## Additional Information and Declarations

### Data Availability

The following information was supplied regarding data availability: The data and code are available in the Supplemental Files.
